# Tool use as adaptation

**DOI:** 10.1098/rstb.2012.0408

**Published:** 2013-11-19

**Authors:** Dora Biro, Michael Haslam, Christian Rutz

**Affiliations:** 1 Department of Zoology, University of Oxford, Oxford, UK; 2 School of Archaeology, University of Oxford, Oxford, UK; 3 School of Biology, University of St Andrews, St Andrews, UK

**Keywords:** technological evolution, ontogeny, culture, cognition, anatomy, social learning

## Abstract

Tool use is a vital component of the human behavioural repertoire. The benefits of tool use have often been assumed to be self-evident: by extending control over our environment, we have increased energetic returns and buffered ourselves from potentially harmful influences. In recent decades, however, the study of tool use in both humans and non-human animals has expanded the way we think about the role of tools in the natural world. This Theme Issue is aimed at bringing together this developing body of knowledge, gathered across multiple species and from multiple research perspectives, to chart the wider evolutionary context of this phylogenetically rare behaviour.

## 1. Introduction

A recent, comprehensive compendium on non-human animal tool-use behaviour [1] lists four phyla (Echinodermata, Arthropoda, Mollusca and Chordata) and nine classes (sea urchins, insects, spiders, crabs, snails, octopi, fish, birds and mammals) as containing tool-using species. Within several of these classes, estimates for the number of independent origins for the behaviour range from one to several tens of events [2]. This broad phylogenetic spread of multiple origins, however, goes hand in hand with overall rarity: tool use has been documented in less than 1% of the animal genera currently identified, and an even smaller percentage of species. The evolutionary events that gave rise to this eclectic distribution must find their ultimate explanation in the benefits that tool use offers to individuals in these species (and their ancestors).

Before examining further the question of adaptive value (i.e. the ultimate, functional explanation that centres on the fitness benefits of the behaviour to the individual), we first recognize that tool use is not a unitary phenomenon—it is not one with easily generalizable features across occurrences. Several authors have distinguished, broadly, between two extremes of tool-use behaviours: those that appear ‘hard-wired’ within a species' behavioural repertoire and that are generally not accompanied by other forms of tool use (referring to these cases as ‘specialist’ [3] or ‘stereotyped’ [2] tool users), and those that appear to be adopted largely through a combination of individual and social learning and that may be one of a suite of tool-use behaviours expressed by the species (‘flexible’ [2] or ‘creative’ [3] tool use). While a behaviour that appears according to a fixed ontogenetic pattern strongly suggests past (and likely present) advantages significant enough for natural selection to fix the behaviour genetically (e.g. [4]), the more flexible (and accordingly often not species-wide) adoption of tool use during individual life histories requires detailed examination of adaptive benefits. Nonetheless, in both cases, the burden of proof remains on showing that tool use indeed raises individual fitness.

While the question of adaptation is undeniably key to understanding the emergence of tool-use behaviours, empirical data on the adaptive significance of tool use are surprisingly scarce. The reasons for this are likely rooted in the difficulties associated with obtaining the required long-term field data on tool-use performance and reproductive success. A study of bottlenose dolphins in Shark Bay, Australia, provided a notable first glimpse of the fitness pay-offs in a species that shows intrapopulation variation in tool-use behaviour [5]. Some dolphins inhabiting the bay use sponges during foraging as protective aids for their rostra—and do so for a large proportion of their feeding time—while others never ‘sponge’. When examining long-term calving records, the authors found no difference in the reproductive output of spongers compared to non-spongers, suggesting that the use of tools did not offer significant fitness benefits (nor, indeed, did it entail added fitness costs). Thus, the behavioural polymorphism appears to be maintained as an evolutionary equilibrium where the fitness returns of tool-assisted and non-tool-assisted foraging lifestyles are equalized. In a more nuanced interpretation, the idea that tool use may offer—at least in the Shark Bay environment—frequency-dependent advantages is a reasonable and stimulating suggestion in need of further investigation. Surprisingly, we are unaware of any other studies that have attempted to document, directly, the fitness consequences of tool-use behaviour. Studies on birds have recently provided compelling, quantitative evidence of the energetic benefits of tool-assisted foraging [6,7], but lacked breeding data to test whether better tool users indeed enjoyed fitness advantages. Perhaps most surprisingly, even among the long-studied chimpanzee populations of Gombe and Mahale in Tanzania and Bossou in Guinea, which have records of tool use and genealogies extending back many decades, no published research has examined the link between tool-use frequency (or competence) and fitness. This, for now, remains a major research challenge in our field.

The difficulty of obtaining data that unambiguously link tool use to reproductive success suggests that we may more fruitfully put our efforts into identifying those aspects of tool use that are promoted, or depressed, by an animal's physical abilities and surroundings. Amassing such contextual information across a wide variety of species is a necessary step towards the ultimate goal of adequately assessing whether or not a given tool-use behaviour is truly adaptive. In broad terms, this information includes: (i) the ontogenetic mechanisms that allow tool use to be reliably, and correctly, assimilated into an individual's behavioural repertoire, including the possible presence of hardwired triggers; (ii) the constraints of an animal's morphology that enable some kinds of tool use and rule out others (e.g. the ability to grasp and manipulate a tool in the rostrum, claw, hand, beak, trunk, mandible or pereiopod); (iii) the extent to which an animal may perceive the need for, and can successfully implement, the problem-solving routine that we observe as tool use (whether or not this is described as a ‘cognitive’ ability); and (iv) the external pressures and opportunities provided by an organism's social and ecological conditions during its life history (e.g. solitary versus group-living systems; terrestrial versus underwater environment; relative profitability of different foraging modes).

The papers in this Theme Issue each address at least one, and typically several, of these four interrelated topics. Collectively, they highlight a broad spectrum of approaches in tackling the costs and benefits of tool use. The contributions cover birds, dolphins, monkeys, apes and both modern and extinct humans—taxa that have each received intense scientific attention for their tool-use behaviour—and feature research from diverse disciplines, including behavioural biology, evolutionary ecology, psychology, neuroscience, anatomy, anthropology and archaeology. The impetus for this collection was a Royal Society International Scientific Seminar held in April 2012, with the same title as this Theme Issue.

## 2. Ecology, society and selection: constraints and facilitators inherent in the environment

Several papers in our Theme Issue examine environmental factors that influence the expression and characteristics of tool-use behaviours, and they do so across different levels of comparison: between biomes [8], between wild and captive conditions [9], between seasons [10] and between environments differing consistently in quality [11].

On the broadest scale, Mann & Patterson [8] consider differences in tool use between aquatic habitats and the better-studied terrestrial environment. While tool use has been recorded in a variety of taxa under water (see table 1 in [8]), it appears even rarer than it is on land. The authors suggest a number of factors that might explain this finding, including limited scientific knowledge due to challenging observation conditions, animals' manipulative limitations due to body plans adapted primarily for streamlining, the fact that the viscosity of water reduces the effectiveness of certain actions (such as pounding), and the lack of available tool materials throughout the water column other than in benthic environments. In terms of qualitative differences, they note that slower decomposition of organic material and the availability of sessile animals have promoted the use of animals (or their products) as tools. Finally, for the best-studied aquatic tool users—dolphins and sea otters—the authors discuss some potential commonalities, such as the observation of individual-level tool-use specialization to extents not often seen in terrestrial systems. This suggests that aquatic environments can provide fertile ground for exploring the fitness benefits of tool use, by presenting case studies where direct comparisons between sympatric tool users and non-tool users of the same species are feasible.

Such differences in the degree of individual tool-use specialization can be promoted through natural variation in individuals' propensity or competence at tool use, as well as through frequency dependence—in other words, the benefits that can be derived from tool use may be contingent upon the presence of non-tool users in the population, and act through, for example, reduced intraspecific competition. Alternatively, variation in the frequency and diversity of tool use expressed by members of the same species may derive from environmental factors—in this case necessarily separating the variation in space or time. Three papers in our issue—all dealing, at various time-depths, with tool use within the hominid radiation—examine such effects and their evolutionary implications.

Haslam [9] discusses the observation, particularly pronounced among the great apes, that individuals in captivity exhibit a greater range of tool-related behaviours than their counterparts in the wild, at least when quantified as the different ‘modes’ [1] of actions, manufacturing and combinatorial processes performed. Referring to this as the ‘captivity bias’, Haslam hypothesizes a number of environmental and social factors that could account for the effect. These include increased free time and increased access to both materials and individuals (including humans) already skilled in using them as tools. The central thesis—that reliance on observations from the wild would therefore tend to underestimate the tool-related cognitive capacities of members of a species—is then extended to the hominin lineage. Haslam points out that for our ancestors and their extinct, close relatives, everything we know about the evolution of cognition is grounded, inevitably, in analyses of changes in cranial anatomy and in the material record produced by tool-making and tool-using activities. With the captivity-bias effect in mind, the latter generates both a problem and potential solution: first, we may be underestimating the full cognitive faculties of these species, but second, precise consideration given to the specific ecological and social circumstances that different hominin groups may have faced should lead to refined estimates of their capacities. Reconstructing the habitats and social dynamics of long-extinct species poses major methodological challenges, but promises fresh insights in the quest to infer natural tool-use performance. In any case, as a first step, producing more refined, quantitative demonstrations of the captivity-bias effect appears to be a research priority.

Looking less far, yet in greater quantitative detail, into the hominin past, Collard *et al*. [11] dissect potential drivers of technological evolution in *Homo sapiens*. Specifically, they examine variation in the complexity of tool-use and tool-manufacturing techniques found among small-scale hunter–gatherer societies, seeking out variables that best predict the observed patterns. The study uses a new dataset covering early contact-period populations in North America (covering the sixteenth to eighteenth centuries) that describes the types and number of constituent components of tools employed by each group. By correlating across populations ‘technological richness’ with measures of various environmental and demographic variables, the authors find tantalizing evidence for the ‘(environmental) risk hypothesis’. This hypothesis states that the use of more specialized, and therefore more elaborate, tools may buffer against the risks of resource failure, leading to richer tool kits in riskier environments. These new results add to those obtained by the same authors analyzing food-producer societies [12], in which tool-kit diversity was more affected by population size, in line with predictions from models of cultural evolution and earlier empirical investigations (e.g. [13,14]). The way in which physical and social environmental variables interact with other parameters (in this case, the population's primary food-getting strategy) to drive technological evolution echoes suggestions in the previous paper [9] that these variables should not be considered in isolation. Collard *et al*.s [11] new findings are likely to spark fruitful debate among researchers interested in human material culture, and may even inspire first tests in some non-human systems that exhibit enhanced levels of tool diversification and complexity.

Finally, studying modern humans' closest living relatives, Sanz & Morgan [10] examine environmental and social parameters as possible drivers behind chimpanzee tool-use frequency, complexity and diversity. In contrast to the multi-population comparison performed by Collard *et al*. [11], Sanz & Morgan study a single community of chimpanzees inhabiting the Goualougo Triangle in the Republic of Congo, but do so in great detail over several years, allowing them to assess seasonal variability. They chart both temporal changes in the relative abundance of food resources (food targeted with and without tools) and in tool-use behaviours, to evaluate empirical support for several different hypotheses that have been put forward to explain (variation in) the expression of tool use. These non-mutually exclusive accounts posit, respectively, that tool use: (i) can serve to compensate for the reduced availability in the environment of foods accessible without tools; (ii) emerges when either tools or high-quality resources in need of tool-assisted processing—i.e. opportunities for tool use—are abundant; (iii) is expressed whenever the relative profitability of tool-assisted foraging exceeds that of alternative foraging techniques; and (iv) can only be maintained if there are sufficient opportunities for observational (social) learning. The chimpanzee data provide no support for the tracking of resource abundance (i) (i.e. scarcity of preferred fruits did not lead to increased tool use), but instead highlight the importance of tool-use opportunities (ii) (i.e. gathering of termites, ants and honey when these were available). Reviewing published evidence from other chimpanzee populations, and other species, the authors find mixed results, emphasizing that hypothesis (iii) effectively encompasses the other two ecological accounts ((i) and (ii)) (as noted previously by Rutz & St Clair [15]) but remains notoriously difficult to test. This encourages a shift in focus towards studying the energetics, and relative profitabilities, of different foraging modes, in different habitats and across seasons. Work like this requires hard-won, longitudinal field datasets, just like the one being generated by the Goualougo Triangle chimpanzee project, but should lead to a much enhanced understanding of the ecological contexts that select for tool-use behaviour.

## 3. Ontogeny: interactions between hard-wiring, external triggers and learning

Trajectories of tool-use development show immense variation across species. Some appear as genetically fixed action patterns, some are acquired through individual learning (in some cases channelled by specific behavioural predispositions) and some are cases of social (or socially scaffolded) learning. Support for the first—or at least for some degree of genetic control—comes from studies that report consistency in the age at which individuals reach developmental milestones during tool-use ontogeny, often even in the absence of those social or environmental inputs that one might suspect to be key triggers (e.g. [4,16,17]). On the other hand, for both individually and socially acquired behaviours (e.g. [18–20]), the physical and/or the social environment must present sufficient opportunities—or sufficient necessity (see [9–11])—to promote individuals' tool-use learning, notwithstanding any possible morphological or cognitive prerequisites. The papers of this section focus on the diverse processes that ensure the predictable development of proficient tool use in a range of taxa.

Chappell *et al.* [21] examine the developmental context of tool ‘invention’ in the world's most flexible tool user, *Homo sapiens*. While previous work has confirmed that even very young children are adept tool users and can distinguish functional from non-functional tools (a theme closely related to enhancing the potential adaptive benefits of tool use), the ability to solve unfamiliar problems through spontaneously creating a new tool emerges relatively late [22]. Yet such transformations must be key to both the diversity and the cumulative sophistication that characterizes human technological evolution. In their series of experiments, Chappell *et al.* study the abilities of 4–7 year-old schoolchildren to devise appropriate modifications of existing tools when given a novel problem, and test whether success is dependent on, or at least facilitated by, demonstrations, verbal prompting, exploration of materials and/or provision of added time to consider the problem. By far the most powerful enhancement is experienced after an explicit demonstration of the tool being transformed; in fact, none of the other potential cues improved performance in the children tested. The authors suggest that the main difficulty derives from the nature of the problem of devising novel tools, in that it involves connecting starting conditions with a desired end-state through material transformations or actions whose specifics are not immediately apparent (the so-called ‘ill-structured’ problem). The study thus sheds light on how cognitive developmental milestones—specifically, in this case, the emergence of the ability to solve ill-structured problems—can contribute to flexibility in tool use and the eventual diversification and increased sophistication of a species' tool kit.

Documenting the development of multiple forms of tool use in two species of primate, chimpanzees and capuchin monkeys, Fragaszy *et al.* [23] highlight how artefacts themselves create rich learning opportunities for young individuals (see also [24])—an observation alluded to in several other papers of our Theme Issue [8,10,25,26]. Encountering objects that others have used as tools, as well as food and tool debris (often in configurations that contain information about the actions and mechanics of the full behavioural sequence), clearly contributes to young primates' learning of tool-related behaviours. The authors confirm robust developmental changes in young's attention to artefacts used, or produced, by fellow group members. The younger the learner the more attractive adult-used objects are, and for behaviours that involve a tool-manufacturing phase, this is typically preceded by a period of exclusively using tools made by others. Framing their ideas in ‘niche-construction theory’ [27], Fragaszy *et al.* argue that, for an individual developing in a tool-using society, the social and physical components of its ontogenetic niche combine in a powerful way to channel tool-related learning. An interesting prediction from this general line of reasoning is that the longevity of the materials that serve as tools, and their likelihood of accumulation, should correlate with the prevalence and persistence of the respective tool-use behaviours. Finally, addressing directly the theme of our issue, Fragaszy *et al.* argue that the characteristics of the learning process observed (attraction to artefacts and subsequent object-guided learning) are themselves adaptive—rather than only tool use itself.

Taking an even broader comparative approach, Meulman *et al*. [25] explore general life-history traits that may promote the adoption of foraging tool use through social learning. They propose that species that show ‘habitual’ foraging tool use in the wild (i.e. tool use that is exhibited routinely by some, but not necessarily all, members of a population [28]) may have in common a propensity to acquire tool use socially, with implications for our understanding of how technological sophistication accumulates at both the individual and the population level. Species that, according to our current state of knowledge [1], fit the definition of habitual foraging tool users include several primates (orangutans, chimpanzees, capuchins and long-tailed macaques), aquatic mammals (bottlenose dolphins and sea otters) and birds (woodpecker finches, New Caledonian crows and green-backed herons). Three lines of evidence are presented that point towards the involvement of both individual and social learning in the maintenance of tool-using skills in given habitually tool-using populations: (i) detailed longitudinal studies of development, which often reveal long periods spent honing the skill (see also [24]); (ii) experimental studies on the cognitive processes that may be underpinning the more flexible forms of tool use often expressed by habitual tool users (see also [26,29–31]); and (iii) observational studies showing how the social environment is able to structure the learning and expression of tool-using skills (see also [10,23]). Whether life-history parameters, such as long dependency periods, indeed promote flexible, habitual tool use remains speculative, especially since evolutionary causality cannot be established without formal comparative analyses. But, given our interest here in the adaptive significance of tool use, further exploration of the link between social learning as a principal transmission mechanism, and the susceptibility of tool-use behaviours to cultural variability, cumulative changes and extinction, is an exciting viewpoint addressed by taking an ontogenetic stance.

## 4. Individual capacities: specificity and flexibility

While ontogenetic studies can illuminate behaviours and cognitive capacities that scaffold the development of tool use, the performance of skilled tool users provides further important clues to the potential lifetime adaptive benefits of the behaviour. Whether or not a specific form of tool use is enabled by advanced cognition, from a cost–benefit perspective we expect selective advantages to accrue if animals are able to select suitable objects as tools, to modify them in ways that improve their efficiency, and to apply them in the appropriate fashion to suitable targets. Two experimental papers in this section focus specifically on animals' ability to discriminate features of objects that make them more or less functional as tools for specific tasks [26,29], while two others provide empirical data [30] and review published literature [31] to make explicit cross-species comparisons. Such comparisons address both the adaptive benefits of tool use in specific circumstances and the notion that certain forms of tool-use behaviour may be dependent on specialized cognitive faculties.

It was only in 2007 that the first scientific account was published of Burmese long-tailed macaques' use of stones to process marine and other prey in intertidal habitats ([32]; see cover image), expanding the catalogue of known stone-tool-using primates from three to four (the others being bearded capuchins, western chimpanzees and humans [33,34]). These monkeys are notable for the variety of food items they process—at current count, 47 different plant and animal species, including oysters, snails, crabs and sea almonds [35]. In their contribution to our Theme Issue, Gumert & Malaivijitnond [29] examine whether long-tailed macaques exhibit prey-specific choice of stone tools. They performed a field experiment involving the presentation of stones of different sizes on the shores of Piak Nam Yai Island in Thailand, and monitored the monkeys' selections as they picked out tools to process prey harvested nearby. Both the results of this field experiment, and of complementary surveys of trace evidence from naturally occurring tool use, confirm that monkeys match stone size to the demands of processing prey of different size and hardness. These findings align with field experimental results from other species, such as capuchin monkeys [36] and New Caledonian crows [26], demonstrating capacities in wild animals to select tools according to size, shape or mechanical properties, in ways that are assumed to increase their effectiveness.

New Caledonian crows are the only non-human animal that has been described to fashion hooked foraging tools in the wild, which they use to fish for prey in deadwood and vegetation [37]. Importantly, the hooked end of tools is functional only if the birds orient their tools correctly during deployment. Noting the clear adaptive significance of orienting hooked stick tools appropriately, St Clair & Rutz [26] conducted a suite of experiments to investigate whether wild-caught crows attended to the functional properties of supplied tools. All subjects indeed paid close attention to which end of a tool was hooked, even when they were presented with replica tools in which features that were normally co-occurring at the tool's functional end (hook; curvature; stripped bark) were experimentally set in conflict. These findings contrast with those of an earlier study, in which wild-caught New Caledonian crows [38] did not appear to attend to the orientation of (natural) barbs on tools made from *Pandanus* spp. leaves. St Clair & Rutz offer a number of explanations for this apparent disagreement, ranging from potential artefacts of experimental design, to differences in the natural manufacturing process of the two tool types. Together with Gumert & Malaivijitnond's field studies [29], these thorough experiments under naturalistic conditions are aimed at better understanding the decision-making processes underlying successful tool selection and deployment. Although some of the results are striking, St Clair & Rutz [26] caution that tool selectivity could be produced by very basic processes (e.g. an evolved neurological predisposition or learning during ontogeny) and does not in itself constitute evidence that animals exhibit ‘causal understanding’ of tool affordances, or possess other advanced cognitive abilities.

Examining related issues, Teschke *et al*. [30] address the role of cognition either as a (probably domain-general) pre-adaptation to flexible tool use or as a (more domain-specific) adaptation that has evolved to support increasingly sophisticated forms of tool use. Through carefully targeted comparative work they examine whether naturally tool-using species possess cognitive capabilities that differ measurably from those of their close, naturally non-tool-using relatives. The same approach has been previously employed in both birds and primates [39–41], including detailed within- and between-species analyses in Darwin's finches by Teschke *et al*. [42]. The present study by this team adds another pair of related species to their earlier work [42], with the same physical-cognition and general-learning tasks presented to both tool-using New Caledonian crows and non-tool-using carrion crows. In this new corvid comparison, but not in the pair of Darwin's finches studied previously, the tool-using species ‘outperforms’ its non-tool-using counterpart on tasks involving physical cognition (but not on those testing general-learning abilities). While the paper openly discusses several reasons for why the results should be treated cautiously, the authors hypothesize that the relative sophistication expressed in tool use by the corvids compared to finches may play a role—the more varied and complex tool use of New Caledonian crows may represent a level of flexibility at which enhanced physical cognition enters either as a driver or as a consequence. Interestingly, the relatively poor performance of New Caledonian crows on some extensions of the original physical-cognition task appears to hint towards varying readiness to attend to different types of perceptual cues, much like the previously discussed observation that tool features may guide effective tool-orientation decisions when handling some tool types, but not others ([38] cf. [26]).

Continuing in a comparative vein, and inspired by some authors' recent reference to corvids as ‘feathered apes’ (a proposal based on the existence of a suite of purportedly similar cognitive capacities; e.g. [43,44]), McGrew [31] performs a direct comparison between New Caledonian crows and chimpanzees, the two non-human species typically considered the most ‘advanced’ animal tool users. Reviewing the vast catalogue of literature on chimpanzee tool use and the growing body of field reports and laboratory studies on New Caledonian crows, McGrew takes stock of species differences and similarities, searching for possible signatures of convergent evolution. Although along some axes of comparison tool use by New Caledonian crows approaches or even surpasses chimpanzee technology (e.g. manufacture of hooked foraging tools [26]), in others the apes register higher counts of observed behaviours. McGrew's particular emphasis is on tool function: while New Caledonian crows use tools primarily for extractive foraging (but see [45]), chimpanzees also employ tools extensively for self-maintenance and in the social domain. Scores are likely to even out further as research on New Caledonian crows continues—with new field experiments (e.g. [26,46]) and increasingly sophisticated technologies for observation [47]—but it remains to be seen whether we are indeed dealing with a case of convergent evolution from which meaningful evolutionary drivers can be gleaned. For detecting general patterns, broader comparative studies—involving multiple primate and bird species, or even more diverse taxonomic samples—represent a challenging but potentially very productive avenue for future research.

## 5. Morphology and the body–tool interface

As tool use becomes ever more tightly engrained in the behavioural repertoire of a species, we may expect to see changes over evolutionary time that reflect its adaptive value through gross morphological changes that represent a better fit to the demands of the tasks, either in the bodies of tool users or in the design of the tools themselves. Humans are by far the most versatile tool users in existence, with all societies reliant daily on a range of tools dedicated to a multitude of purposes. In this section, we examine the drivers and consequences of this most extreme case of technological evolution through changes in anatomy, brain organization and tool design.

Papers from both anatomical [48] and comparative neuroscientific perspectives [49] reveal evolutionary changes that inform us about the causal relationships and the ultimate long-term effects of tool technology on human biology. Our final contribution [50] examines the animal and hominin archaeological record for evidence of deliberate orientation and imposition of a ‘long-axis’ on tools, with some tantalizing suggestions that exaggerated design can allow objects manufactured for a specific mechanical purpose to assume novel roles within the symbolic realm.

The advent of stone-tool use was undoubtedly a key event in our own lineage's evolution, eventually leading to the establishment of humans as the most successful tool users on the planet. Already, the earliest known stone tools underwent a manufacturing process that required an accurate balance between precision and strength [51]. Observations of present-day stone-tool makers can reveal a great deal about the specific technical demands of the task, the types of grips and strikes involved and the muscles that enable the correct forces to be exerted [52,53]. Marzke's [48] pioneering analyses of the evolution of the human hand, presented together with comparative data from extant non-human primates, reveal features for grip and stress-accommodation that are necessary to support stone-tool manufacture. Determining whether these derived features indeed evolved specifically in response to tool making will require further work, and may be a significant finding given that gross morphological adaptations to tool use appear only evident in a handful of species [54–58]. Nonetheless, among the most fascinating implications of Marzke's work is its potential to identify specific signatures of skilled stone-tool manufacture that can serve as diagnostics for fossil hominins, including numerous australopithecines and *Homo* spp., whose tool-making capacities are currently unknown.

Apart from such gross anatomical changes associated with the adoption of tool use, it may also be hypothesized that the behaviour may be accompanied by at least some degree of reorganization in the brain (cf*.* [30]). The scale of such changes may vary and may take place over both developmental and evolutionary timescales. In their work on the former, Iriki *et al*. [59] and Maravita *et al.* [60] have provided demonstrations that, following experience with a specific tool-using task, the brains of both humans and monkeys come to perceive tools as extensions of the individuals' bodies. Looking over evolutionary time, but using a similar approach that entails recording how parts of the body are mapped onto the brain, Hashimoto *et al*. [49] present in our Theme Issue comparative neuroimaging data that address a long-standing conundrum in human evolution: the relative timing of the appearance of bipedalism and tool use among our ancestors. Did the shift to bipedalism act as a catalyst for tool use by *first* freeing up the hands, or did the adoption of tool use encourage the shift to upright gait *in order to* free the hands from locomotion? By recording primary sensorimotor cortex responses to stimulation applied to individual fingers and toes in both monkeys and humans, the authors report an interesting difference between the two species in their somatotopic representation of the digits. While both species represent fingers as separate units, suggesting a likely adaptation to manual dexterity shared across the primate lineage, the mapping of the human foot appears to possess a derived feature. The fused representation of all five toes, as it occurs in monkeys, has been replaced in humans by a dedicated somatotopic representation of the big toe separate from the other four—a change hypothesized to be associated with the shift to bipedalism. Comparative data, which the authors supplement with fossil evidence, thus suggest that the manual dexterity supporting tool use had already appeared in the primate lineage prior to any significant changes in locomotion among hominins, and thus that neurological control of tool use among our ancestors was not solely driven by the evolution of bipedalism.

As tools represent the direct interface between the animal and its environment, their design aspects deserve attention in themselves as indicators of effectiveness and adaptation. Gowlett [50] focuses on one particular aspect of multivariate tool design: elongation. Defined as extending the length of an object in relation to its width, elongation produces tools that serve a variety of purposes, and involves the imposition of discrete, use-related, axes on a material object. Elongated tools are found both within the hominin line and among non-human animals (including the types of stick tools manufactured and used by chimpanzees [10] and New Caledonian crows [26] in our volume). Several questions are addressed by Gowlett's review of the distribution and prevalence of elongated artefacts within the hominin Acheulean tradition. For example, he suggests that elongation was often unlikely to have been an end in itself, but instead represented one end of a continuum of shapes that serve specific needs in different tasks. Intriguingly, it is possible that the excessive application of this feature (beyond extents that actually improve the tool's effectiveness, such as in the case of oversized or ‘overfinished’ tools) signals the appearance of a symbolic significance to tool making—in other words, the time when skilled tool-making comes to represent adaptation from a different (sexual) selective viewpoint. Gowlett identifies valuable opportunities to conduct comparative work on human and non-human animal tools, which may reveal the relative contributions to tool manufacture of material, artefact or task constraints versus abilities to plan and execute technical steps involved in transforming objects into tools.

## 6. Conclusion

The ideas collated in this Theme Issue come from researchers working in diverse disciplines, including psychology, ethology, archaeology, ecology, neuroscience and anatomy. We believe that continued, and increased, collaboration between specialists from these fields is required as we home in on answering fundamental questions about tool use. This said, the fact that the definition of tool use itself is still being revised and debated (e.g. [61]) indicates that we have some way to go before we can say that we know *why* animals use tools, and why humans became so dependent on them. But, without input from the varied fields represented in this volume we are unlikely to resolve these issues at all. A critical addition to this point is that we need high-quality research data from many more tool-using species: studies that aim to identify commonalities and differences between groups or species—in terms of ecological drivers, general cognitive or morphological prerequisites, or the role of social learning—depend on such comparative data.

We have stressed here the role of adaptation (i.e. reproductive advantage) as an ultimate explanation for tool use, acknowledging the fact that we cannot identify adaptations without first qualifying, and where possible quantifying, the variation on which natural selection may act. By taking such a broad approach, we hope that our volume has succeeded in taking us a step closer to understanding tool use as adaptation.
